# Hybrid rye grain inclusion strategies as a replacement for corn grain in finishing cattle diets: effects on growth performance and carcass characteristics

**DOI:** 10.1093/tas/txaf059

**Published:** 2025-05-02

**Authors:** Federico Podversich, Warren Rusche, Scott Bird, Brad Rops, Pete Sexton, Zachary Smith

**Affiliations:** Department of Animal Science, South Dakota State University, Brookings, SD 57007, USA; Department of Animal Science, South Dakota State University, Brookings, SD 57007, USA; South Dakota State University Agricultural Experiment Station, Southeast Research Farm, Beresford, SD 57004, USA; South Dakota State University Agricultural Experiment Station, Southeast Research Farm, Beresford, SD 57004, USA; Department of Agronomy, Horticulture and Plant Science, Brookings, SD 57007, USA; Department of Animal Science, South Dakota State University, Brookings, SD 57007, USA

**Keywords:** beef cattle, feedlot, hybrid rye, phase feeding, winter cereal

## Abstract

Two experiments were conducted to evaluate the effects of feeding dry-rolled hybrid rye grain (DRRG) as a replacement for dry-rolled corn (DRC) in beef cattle finishing diets. Two inclusion strategies for rye grain (RG) were evaluated: a total replacement of DRC for a limited time and a partial replacement during the entire feeding trial for Exp. 1 and 2, respectively. In Exp. 1, primarily Angus steers [n = 110, initial shrunk body weight (BW) 391 ± 31.5 kg] were blocked by BW and pen location in 7 blocks and assigned to either 1-A) DRC as the sole grain during the entire finishing (DRC), or 1-B) DRRG during the initial 46 d (replacement phase) and then switched to DRC (DRRG/DRC). In Exp. 2, primarily Angus steers (n = 44, initial shrunk BW 436 ± 41.0 kg) were blocked by BW and pen location in 4 blocks and assigned to either 2-A) DRC as the sole grain (DRC), or 2-B) the dietary grain component was a blend of one-third DRRG and two-thirds DRC (DM basis), during the entire feeding trial (MIX). Steers were fed for a total of 144 and 119 d in Exp. 1 and Exp. 2, respectively. Pen was the experimental unit, and data were analyzed as a randomized complete block design for both experiments. In Exp. 1, DRRG-fed steers had 18% less dry matter intake (DMI; *P* = 0.01) and 13% less average daily gain (ADG; *P* = 0.01) during the replacement phase. Cumulatively, steers initially fed with the DRRG-based diet tended to consume less than steers continuously fed the DRC-based diet (*P* = 0.08). However, cumulative ADG, final body weight (FBW), and hot carcass weight (HCW) did not differ (*P* ≥ 0.16). Steers fed DRRG tended to have lesser marbling scores and USDA Yield Grades (*P* = 0.08). In Exp. 2, Steers fed MIX tended to have less DMI expressed as % of BW (*P* = 0.09); no other differences were observed for growth performance or carcass characteristics. In conclusion, both alternatives serve to include rye in feedlot diets, with minimal effects on cumulative performance. However, in the current experiment total replacement of DRC with DRRG for a portion of the feeding period reduced feed intake, body weight gain, and carcass quality.

## Teaser

Rolled rye grain can replace corn grain in finishing cattle diets, either one-third of corn grain during the entire feeding or as a total replacement for a limited time. However, total replacement for a portion of the feeding period may reduce intake, gain, and carcass quality, while one-third replacement over the entirety of the finishing period does not affect these parameters.

## INTRODUCTION

Corn is the primary feed grain used in beef cattle finishing systems throughout the United States for several reasons. It produces more metabolizable energy per unit of land than any other alternative crop, and the infrastructure, production strategies, and marketing channels for corn are well-established. However, its popularity should not be confused with perfection. Relying on monocultures or two-crop rotations increases the risk of adverse environmental conditions and pest pressures. Diversifying crop rotations can help break pest cycles and distribute labor requirements more evenly (Poffenbarger et al., 2017; Bowles et al., 2020).

Cereal winter rye (RG) is a strong candidate for a potential rotational crop. However, the presence of ergot alkaloids (EA) in RG is a concern. Rye and triticale are the most susceptible winter grains to infection by Claviceps spp., (the fungi responsible for the synthesis of EA), mainly because their flowers are cross-pollinated, whereas wheat, barley, and oats are self-pollinated (Gaudet et al., 2000; WHO, 2006; Menzies & Turkington, 2015). Recent introductions of hybrid germplasm have shown greater yield potential and improved resistance to ergot compared to open-pollinated varieties (Hansen et al., 2004; Coufal-Majewski et al., 2016; Zhang et al., 2024a). During times of the year when the milling or distillery industries are saturated, incorporating RG into beef cattle diets could serve as an alternative use for this grain. However, for RG to gain broader adoption, more information about its feed value for beef cattle is required.

Our previous studies conducted at South Dakota State University indicate that hybrid RG can replace corn grain in finishing diets (Rusche et al., 2020; Buckhaus et al., 2021). Still, there are considerations for its use to ensure performance is not affected. Rusche et al. (2020) fed diets in which dry-rolled corn (DRC) constituted 60% of diet dry matter (DM) and was replaced with dry-rolled hybrid RG (DRRG; KWS Bono; KWS Cereals LLC, Champaign, IL) at four levels: 0%, 33%, 66%, and 100%. In this study, increasing the replacement of DRC with DRRG linearly decreased dry matter intake (DMI), average daily gain (ADG), and feed conversion (Rusche et al., 2020) with negligible differences between 0% and 33% replacement. Additionally, there were no differences in dry matter intake (DMI) between treatments for the first 56 d with reduced DMI for 66 or 100% replacement not occurring until later in the feeding period (Rusche et al., 2020). As a result of these previous studies, it appears that hybrid RG can replace approximately one-third of dry-rolled corn in finishing beef cattle diets without negatively impacting growth performance or carcass value. Including hybrid RG in greater proportions reduced final performance, but there were no differences in DMI or ADG for the first 56 d.

These observations led us to consider two strategies for the use of hybrid DRRG in finishing diets. The first option would be to replace all DRC with hybrid DRRG as the sole grain source during the initial 6 wk then switch to DRC. The second option would be to replace approximately one-third of the DRC with hybrid DRRG during the entire finishing period. These strategies could enable producers to use corn and RG in proportions that closely match the combined yield of each crop. In addition, this flexibility would allow cattle feeders to take advantage of temporary price differences between rye and corn to reduce the cost of gain.

Our objectives for these two experiments were to determine the effects of hybrid DRRG inclusion as a complete replacement for DRC during the initial phase of the finishing diet or as a partial replacement during the entire finishing period on DMI, growth performance, and feed efficiency in finishing beef steers and to estimate net energy (NE) value. Our hypotheses were that for Exp. 1. hybrid DRRG can replace DRC in finishing beef diets during the initial 46 d of feeding without affecting the cumulative growth performance or carcass traits at the end of the finishing trial; and for Exp. 2 hybrid DRRG can replace 31.8% of the DRC in a finishing beef diet during the entire trial, without affecting the cumulative growth performance or carcass traits.

## MATERIAL AND METHODS

### Institutional Animal Care and Use Approval

Experiment 1 was conducted at the Southeast Research Farm (SERF) in Beresford, SD between April 2023 and September 2023. The animal care and handling procedures used in this study were approved by the South Dakota State University Animal Care and Use Committee (Approval Number: 2303-033E).

Experiment 2 was conducted at the SERF between October 2023 and February 2024. The animal care and handling procedures used in this study were approved by the South Dakota State University Animal Care and Use Committee (Approval Number: 2309-083E).

### Dietary Treatments

The analyzed nutrient composition of feed ingredients is presented in Table 1. Individual ingredient samples were collected weekly and analyzed for DM [method no. 935.29 (AOAC, 2012)]. Weekly ingredient samples were composited by month and analyzed for nitrogen [method no. 968.06 (AOAC, 2016); Rapid Max N Exceed, Elementar Americas, Inc., Ronkonkoma, NY], and ash [method no. 942.05 (AOAC, 2012)]. Because grains are minor contributors of neutral detergent fiber (NDF) to test diets, NDF was estimated to be 9.7% and 15.4%, for DRC and RG, respectively; fiber content analysis for all other ingredients was conducted as described by Goering and VanSoest (1970). Modified distillers grains with solubles (MDGS) were analyzed for ether extract (EE) using an Ankom Fat Extractor (XT10; Ankom Technology, Macedon, NY); tabular EE values were used for the remainder of the ingredients (NASEM, 2016). Net energy values shown in Table 1 are tabular estimates according to Preston (2017).

**Table 1.** Analyzed chemical composition of the feed ingredients^1^ utilized in both experiments

					% of DM	
Item	DM, % of as fed	Ash	CP	NDF	ADF	EE
Sorghum silage	29.4	5.54	7.22	51.07	27.05	--
Dry-rolled Corn	89.6	1.51	8.66	--	--	--
Hybrid rye grain	90.9	2.08	13.95	--	--	--
Modified Distillers Grains	50.2	6.13	33.41	28.28	6.57	7.69
Brome grass hay	89.4	8.40	12.00	65.50	42.00	--
Liquid supplement	65.0	37.68	26.90	--	--	0.21

^1^CP: Crude protein; NDF: neutral detergent fiber; ADF: acid detergent fiber; EE: ether extract.

In Exp. 1, predominately Angus steers [n = 110, initial shrunk body weight (BW) 391 ± 31.5 kg] were assigned to 1 of 14 pens (35 m × 14 m open lot dirt pens with 6 m linear bunk space and 3 m concrete apron; 7 or 8 steers/pen) in a randomized complete block design [blocked by initial body weight (BW) and pen location within the feedyard]. Pens were randomly assigned to one of two treatments (7 pens/treatment): DRC as the sole grain source during the 144 d of feeding **(DRC)** or DRRG as the sole grain source during the first 46 d of feeding followed by DRC as the sole grain for the remaining days of the experiment **(DRRG/DRC)**. Steers were transitioned to a high-grain diet using either DRC or DRRG, depending on the treatment over a 14-d period. Diets fed during Exp. 1 are presented in Table 2.

**Table 2.** Dietary composition and formulated concentration of nutrients, and energy content of the diets fed to beef steers (Exp. 1)

Item	Treatment^1^ diet phase					Common diet phase			
	d 0 - 7		d 8 - 14		d 15 - 46	d 47 - 91	d 92 -144		
	DRC	DRRG	DRC	DRRG	DRC	DRRG		DRC	DRC
*Ingredients, % DM basis*									
Dry-rolled corn	45.7	--	56.6	.	66.1	.		66.0	63.1
Dry-rolled hybrid rye grain	--	45.4	.	56.9	.	66.5		.	.
MDGS^2^	20.5	20.6	19.6	19.5	20.1	19.8		20.2	23.6
Sorghum silage	4.4	4.4	4.0	4.0	3.9	3.8		3.9	9.5
Brome grass hay	25.5	25.7	15.8	15.6	6.0	6.0		6.0	.
Liquid Supplement^3^	3.9	3.9	4.0	4.0	3.9	3.9		3.9	3.8
* Nutrient composition* ^4^									
DM, % as fed	72.4	71.9	70.1	70.5	71.1	71.1		71.8	68.3
OM, % of DM	94.2	93.9	94.9	94.6	95.6	95.2		95.6	96.5
CP, % of DM	15.2	17.7	14.7	17.7	14.5	17.9		14.5	15.1
NDF, % of DM	29.2	31.9	23.4	26.5	18.0	21.7		18.0	17.6
NEm, Mcal/kg DM	1.89	1.74	1.98	1.80	2.07	1.87		2.07	2.08
NEg, Mcal/kg DM	1.20	1.08	1.29	1.14	1.38	1.21		1.38	1.39
Total Ergot Alkaloids, mg/kg	--	0.24	--	0.30	--	0.35		--	--

^1^From d 1 to d 46, pens were fed either a dry rolled corn grain-based- (DRC) or a dry rolled rye grain-based diet (DRRG), depending on treatment assignment; after d 46, all pens received the DRC diet.

^2^MDGS: Modified distillers grains with solubles.

^3^Provided 30 g/ton of monensin as well as vitamins and minerals to exceed requirements (NASEM, 2016).

^4^Dry matter was measured weekly, nutrient composition and ergot alkaloid concentration were analyzed from composite ingredient samples. Net energy vales were estimated from Preston (2017). Dry matter (DM), organic matter (OM), crude protein (CP), neutral detergent fiber (NDF), net energy for maintenance (NEm), net energy for gain (NEg).

Experiment 1 evaluated the effects of total replacement of corn grain with hybrid RG in a finishing diet during the initial 46 d of feeding followed by a DRC-based diet on growth performance and carcass characteristics. The experimental period for the phase feeding trial (Exp. 1) consisted of 114–d of growth performance evaluation (days 0 to d 144). Until d 46 of the experiment, steers were fed either a DRC-based or DRRG-based diet; beginning on d 47 all steers were fed a common DRC-based diet.

Steers were fed once daily at 0800 hours, and bunks were managed according to a slick bunk management system that allowed ad libitum access to feed with minimal day-to-day variation in feed deliveries. Feed was manufactured in a commercial mixer wagon (6.1 m^3^; Reel Auggie 3120, Kuhn North America, Inc., Brodhead, WI) with a scale resolution of 0.91 kg.

In Exp. 2, predominately Angus steers (n = 44, initial shrunk BW 436 ± 41.0 kg) were assigned to 1 of 8 pens (12.15 m × 4.5 m partially covered concrete-floor pens with 4.5 m linear bunk space; 4 or 5 steers/pen) in a randomized complete block design (blocked by initial BW and pen location).

This experiment evaluated the partial replacement of a corn grain with hybrid RG in a finishing diet during the entire feeding period. Diets fed during Exp. 2 are presented in Table 3. Pens were randomly assigned to one of two dietary treatments (4 pens/treatment):

**Table 3.** Dietary composition, formulated concentration of nutrients, and energy content of the diets fed to beef steers (Exp. 2)

	Adaptation^1^ diet (d-21 to-1)			Treatment^2^ diets (d 0 to 119)	
	Week 1	Week 2	Week 3	DRC	MIX
* Ingredients* ^2^ *, % DM basis*					
Dry-rolled corn	51.9	59.1	67.5	67.5	46.1
Dry-rolled hybrid rye grain	.	.	.	.	21.4
MDGS^2^	16.0	17.2	18.3	18.7	18.7
Sorghum silage	20.8	19.7	10.2	9.9	9.9
Brome grass hay	7.2	.	.	.	.
Liquid Supplement^3^	4.1	4.0	4.0	3.9	3.9
* Nutrient composition* ^4^					
DM, % as fed	56.8	57.5	67.6	64.7	64.4
OM, % of DM	94.9	95.5	95.8	95.8	95.7
CP, % of DM	13.3	13.4	13.8	13.9	15.0
NDF, % of DM	24.9	20.7	16.9	16.9	18.1
EE, % of DM	3.35	3.40	3.48	3.39	3.49
NEm, Mcal/kg DM	1.90	1.98	2.07	2.06	2.00
NEg, Mcal/kg DM	1.22	1.30	1.38	1.37	1.33
Total Ergot Alkaloids, mg/kg	.	.	.	.	0.11

^1^Steers from both treatments received the same diet during the step-up adaptation (3 wk) prior to the start of the trial.

^2^For 119 d, steers received either a dry-rolled corn grain-based diet (DRC) or a mix of dry-rolled rye and corn-based diet (MIX).

^2^MDGS: Modified distillers grains with solubles.

^3^Provided 30 g/ton of monensin as well as vitamins and minerals to exceed requirements (NASEM, 2016).

^4^Dry matter was measured weekly, nutrient composition and ergot alkaloid concentration were analyzed from composite ingredient samples. Net energy vales were estimated from Preston (2017). Dry matter (DM), organic matter (OM), crude protein (CP), neutral detergent fiber (NDF), net energy for maintenance (NEm), net energy for gain (NEg).

2-A) DRC as the sole grain source of the diet (DRC)

2-B) a formulated blend of one-third DRRG and two-thirds DRC as the grain portion of the diet, fed during the entire feeding trial (MIX).

The experimental period for Exp. 2 consisted of a 21–d adaptation period (d-21 to d-1), followed by a 119-d growth performance period (d 0 to d 119). During the adaptation, steers from both treatment groups were transitioned to a high-grain diet with the same DRC-based diet for 21 days (d -21 to d 0).

The hybrid RG used for both experiments (KWS Bono; KWS Cereals, LLC) was obtained from SERF. Rye was processed using a roller mill (Lone Star Enterprises, Lennox, SD). The rolls were 23 × 30 cm with 4.7 corrugations per cm in a round bottom v pattern and ran at 857.5 rpm at a 1:1 ratio. Corrugations in one roller were straight, while the second roller was machined with a 12.7-cm spiral design. Rolls were adjusted to achieve a processing index (PI) of 78 as described by Rusche et al., 2020 where PI was defined as the volume weight (g/L) of the grain (as is) after processing as a percentage of the volume weight before processing. The RG was analyzed for EA using liquid chromatography/mass spectrometry/mass spectrometry (North Dakota State University Veterinary Diagnostic Lab, Fargo, ND). Total EA concentration for the RG was 0.526 mg/kg. Ergot alkaloid concentrations for diets containing RG were 0.35 and 0.11 mg/kg for Exp. 1 and 2, respectively.

### Animals, Initial Processing and Study Initiation

#### Experiment 1.

Steers were sourced from a South Dakota auction facility and delivered to SERF. At study initiation (d 0), steers were vaccinated against viral respiratory diseases (Bovi-Shield GOLD 5, Zoetis, Parsippany, NJ) and clostridial species (Ultrabac 7/Somubac, Zoetis). In addition, an appropriate dose of pour-on moxidectin (Cydectin, Elanco Animal Health, Greenfield, IN) was applied, individual BW for allotment purposes was recorded, and steers were identified with a unique visual ear tag. Steers were implanted with 200 mg trenbolone acetate and 28 mg estradiol benzoate (SYNOVEX Plus, Zoetis) on d 19 of the experiment.

The steers’ initial BW was recorded on April 20, 2023, d 46 BW on June 5, and the final BW (d 144) on September 11, 2023. Intermediate BW were obtained on May 9 and August 7, 2023. All live BW measures were pencil shrunk 4% to account for digestive tract fill, in accordance with equations to determine shrunk body weight from NASEM (2016).

Steers were harvested when visually appraised by trained observers to have 12^th^-rib fat thickness of 1.4 cm (RF). Body weights were recorded beginning at 0800 h on the day of shipment, approximately 24 h after feed delivery the prior day. After all steers were weighed, they were returned to their home pens and offered 40% of the previous day’s feed delivery as a method to avoid dark cutting carcasses. Steers were gathered from their home pens at approximately 1300 h prior to delivery to the commercial abattoir located 211 km from SERF. Electronic ID tags were used to determine harvest order. Hot carcass weights, liver abscess scores, and video camera image carcass data were collected from the beef plant.

#### Experiment 2.

Steers were sourced from a SD auction facility and delivered to SERF. Steers were vaccinated against viral respiratory diseases (Bovi-Shield GOLD 5, Zoetis, Parsippany, NJ) and clostridial species (Ultrabac 7/Somubac, Zoetis) on d-21. In addition, an appropriate dose of pour-on moxidectin (Cydectin, Elanco Animal Health, Greenfield, IN) was applied, individual BW for allotment purposes was recorded, and steers were identified with a unique visual ear tag. Steers were implanted with 200 mg trenbolone acetate and 28 mg estradiol benzoate (SYNOVEX Plus, Zoetis) on d 0 of the experiment.

The steers’ initial BW was recorded on October 16, 2023, and the final BW (d 119) on February 12, 2024. Intermediate BW was obtained on November 13, 2023. All live BW measures were pencil shrunk 4% to account for digestive tract fill. Bunk management, feed delivery, feed mixing procedures were identical to those used in Exp. 1. Steers were shipped for harvest when visually appraised by trained observers to have 12^th^-rib fat thickness of 1.4 cm (RF). Final BW were measured using procedures similar to Exp. 1 and steers were shipped 211 km to the same commercial abattoir.

### Growth Performance Calculations

Growth performance (live and carcass-adjusted) for both experiments was calculated on a deads and removals excluded basis. All steers were weighed individually at processing. Steers were weighed in a hydraulic squeeze chute mounted on top of load cells (scale readability ± 0.91 kg). All BW data were shrunk 4% to account for gastrointestinal tract fill. Average daily gain (ADG) was calculated as the difference between initial and final shrunk BW, divided by the days on feed, for each interim period. Cumulative ADG was calculated using carcass-adjusted final BW (hot carcass weight/0.625). Carcass adjustment factor (0.625) matches benchmark data from cattle fed in the Upper Midwest (SD, MN, IA, and NE) in facilities where cattle were exposed to outdoor environmental conditions (Dahlke et al., 2021). Feed conversion efficiency (G:F) was calculated from ADG/DMI.

### Dietary Net Energy Utilization Calculations

For both experiments, observed dietary net energy (NE) was calculated from daily energy gain (EG; Mcal/d) according to the medium frame steer calf equation using the equivalent BW adjustment [standard reference BW = 478 kg and BW of steers at 28.6% empty body fatness (EBF) and was assumed to be equal to adjusted final BW at 28% EBF (AFBW) described by Guiroy et al., 2002]: EG, Mcal/d = ADG^1.097^ × 0.0557W^0.75^; energy gain was the daily deposited energy and W was the mean equivalent shrunk BW (NASEM, 1996). Maintenance energy required (EM: Mcal/d) was calculated by the following equation for both experiments: EM, Mcal/d = 0.077BW^0.75^ (Lofgreen and Garrett, 1968; NASEM, 2016) where BW represented the mean of initial shrunk and final BW.

The observed dietary net energy for maintenance and gain (NE_m_ and NE_g_, respectively) values of the diet were generated using the quadratic formula: x= , where x= NEm, Mcal/d, *a* = -0.41EM, *b* = 0.877EM = 0.41DMI + EG, *c* = -0.877DMI, and NE_g_ = 0.877NE_m_—0.41 (Zinn and Shen, 1998; Zinn et al., 2008). Observed-to-expected (O:E) NEm and NEg were calculated from observed dietary NE values for maintenance or gain divided by tabular estimates of NE for maintenance or gain (Preston, 2017).

### Carcass Trait Determination

For both experiments, hot carcass weight (HCW) was captured immediately following the harvest procedure. Video image data were obtained from the commercial abattoir for rib eye area (REA), rib fat (RF), and USDA marbling scores. Kidney, pelvic, and heart fat (KPH) percentage was determined via plant-specific algorithm. Dressing percentage (DP) was calculated as: HCW/(final BW × 0.96). Yield grade (YG) was calculated according to the USDA regression equation (USDA, 2017). Estimated empty body fat percentage and AFBW were calculated from observed carcass traits (Guiroy et al., 2002). Liver scores were determined by a trained technician and classified according to the Elanco Liver Scoring System (Brown and Lawrence 2010): normal (no abscesses), A- (1 or 2 small abscesses or abscess scars), A (2 to 4 well-organized abscesses less than 2.54 cm in diameter), or A + (1 or more large active abscesses greater than 2.54 cm in diameter with inflammation of surrounding tissue).

### Statistical Analysis

Both experiments were analyzed using the GLIMMIX Procedure of SAS 9.4 (SAS Institute Inc., Cary, NC) using analysis of variance appropriate for a randomized complete block design with pen considered the experimental unit. The model included the fixed effects of treatment and block for both Exp. 1 and Exp. 2. For the distribution of categorical variables (distributions of USDA Yield and Quality grade, and liver abscess incidence and severity), counts for each category were entered by pen, and a multinomial analysis for ordinal data was conducted following the procedure recommended by Bowley (2015), using pen within treatment as the subject. Significance was declared at *P* ≤ 0.05, and tendencies were considered when 0.10 > *P* > 0.05.

## RESULTS

### Experiment 1

Because dietary treatments were applied from d 0 to 46, performance results will be presented by phase: rye feeding period (d 0 to 46), common diet (d 47 to 144), and cumulative performance (d 0 to 144).

Growth performance data for Exp. 1 are shown in Table 4. During the RG feeding phase, steers fed with the DRRG-based diet consumed 18% less feed (*P* = 0.01; 10.30 vs. 8.45 kg/d, for DRC vs. DRRG, respectively) and gained 13% less weight (*P* = 0.01; 1.83 vs. 1.59 kg/d, for DRC vs. DRRG, respectively) than DRC-fed steers. No differences were observed between groups for G:F (*P* = 0.15). Performance adjusted dietary NEm and NEg (paNEm and paNEg, respectively) were 8.6 and 11.2 % greater (*P* = 0.03) for the DRRG-fed steers. Similarly, the ratio of O/E for NEm and NEg differed between treatments (*P* = 0.01). In the case of DRC-fed steers, O/E were 0.98 and 1.00 for NEm and NEg, respectively, while for the DRRG-fed steers, the ratios of O/E for NEm and NEg were 1.18 and 1.26, respectively. During the common diet phase (d 47 to 144), no differences were observed for any of the growth performance variables (*P* ≥ 0.38). Cumulatively, steers fed hybrid RG during the first 46 d tended to consume less feed than those fed DRC-during the entire trial, either expressed in absolute terms (*P* = 0.08; 11.1 vs. 10.6 kg/d, for DRC vs. DRRG, respectively) or as a percentage of BW (*P* = 0.10; 2.19 vs. 2.12 % of BW, for DRC vs. DRRG, respectively). However, no differences were observed (*P* ≥ 0.19) for FBW, carcass-adjusted FBW, ADG, G:F, paNEm, and paNEg. The ratio of O/E for NEm and NEg differed between treatments (*P* = 0.05). For steers that received the DRC-based diet during the entire study, the ratio of O/E for both NEm and NEg was 0.93, while for the steers that were fed initially with the DRRG-based diet, the ratios of O/E for NEm and NEg were 0.97 and 0.98, respectively.

**Table 4.** Growth performance of finishing steers fed either a dry-rolled corn-based diet or a dry-rolled rye-based diet for the first 46 d, followed by a common dry-rolled corn-based diet (Exp. 1)

	Treatment^1^			
*Item*	DRC	DRRG/DRC	SEM^2^	*P*-value^3^
Steers, n	55	54	-	-
Pens, n	7	7	-	-
Initial body weight (BW), kg	391	391	0.1	-
*Treatment phase (day 0 to 46)*				
Average daily gain (ADG), kg	1.83	1.59	0.03	0.01
Dry matter intake (DMI), kg/d	10.30	8.45	0.18	0.01
Gain to feed (G:F; ADG/DMI)	0.178	0.189	0.0049	0.15
Feed to Gain (F:G: 1/G:F)	5.62	5.29	-	-
paNEm^4^, Mcal/kg	1.99	2.16	0.059	0.03
paNEg^5^, Mcal/kg	1.34	1.49	0.052	0.03
Observed/Expected (O/E) NEm	0.98	1.18	0.031	0.01
O/E NEg	1.00	1.26	0.042	0.01
*Common phase (d 47 to 144)*				
d 46 BW, kg	474	464	1.54	0.01
ADG, kg	1.52	1.53	0.04	0.81
DMI, kg	12.4	12.5	0.23	0.61
G:F, kg/kg	0.123	0.122	0.0025	0.84
F:G, kg/kg	8.13	8.2	-	-
paNEm^4^, Mcal/kg	1.78	1.75	0.033	0.38
paNEg^5^, Mcal/kg	1.15	1.13	0.029	0.38
O/E NEm	0.86	0.84	0.015	0.38
O/E NEg	0.83	0.81	0.021	0.38
* Cumulative (day 0 to 144)*				
FBW, kg	623	613	4.8	0.21
Carcass adj. FBW^4^, kg	633	627	5.9	0.37
ADG, kg	1.61	1.55	0.033	0.19
DMI, kg	11.1	10.6	0.16	0.08
DMI, as % of BW	2.19	2.12	0.027	0.10
G:F, kg/kg	0.146	0.146	0.0024	0.93
F:G, kg/kg	6.85	6.85	-	-
paNEm^5^, Mcal/kg	1.92	1.94	0.031	0.58
paNEg^6^, Mcal/kg	1.27	1.29	0.027	0.58
O/E NEm^7^	0.93	0.97	0.015	0.05
O/E NEg^7^	0.93	0.98	0.05	0.05

^1^Treatments were applied from d 1 to d 46. DRC: received the DRC diet during the entire study; DRRG/DRC: received the DRRG diet during the initial 46 d, then switched to the DRC diet.

^2^Standard Error of the mean (n = 7 pens/treatment).

^3^Observed significance for the effect of treatment.

^4^FBW calculated using hot carcass weight divided by 0.625.

^5^Performance calculated Net Energy for maintenance (Zinn et al., 2008).

^6^Performance calculated Net Energy for gain (Zinn et al., 2008).

^7^Observed to expected ratio for NEm and NEg; ratio of paNE values to tabular NE estimates from Preston (2017).

Carcass trait data for Exp. 1 are presented in Table 5. Feeding a DRRG-based diet during the first 46 d of finishing tended to reduce the marbling score (*P* = 0.08; 470 vs. 447, for DRC vs. DRRG, respectively) and USDA Yield Grade (*P* = 0.08; 2.73 vs. 2.57, for DRC vs. DRRG, respectively). No differences were observed between treatment groups for HCW, DP, REA, RF, EBF, and AFBW (*P* ≥ 0.16). Furthermore, no differences were observed between treatments for the distributions of USDA Yield or Quality Grades, nor liver abscess prevalence or severity (*P* ≥ 0.20).

**Table 5.** Carcass traits of beef steers fed either a dry-rolled corn-based diet or a dry-rolled rye-based diet for the first 46 d, followed by a common dry-rolled corn-based diet (Exp. 1)

	Treatment^1^			
	DRC	DRRG/DRC	SEM^2^	*P*-value^3^
Hot carcass weight, kg	397	391	2.7	0.16
Dressing percentage	63.6	63.9	0.40	0.68
Ribeye area, cm^2^	90.3	91.0	1.41	0.71
Rib fat, cm	1.41	1.38	0.052	0.73
Marbling score^5^	470	447	8.6	0.08
Yield Grade	2.73	2.57	0.070	0.08
Empty body fat^7^, %	30.8	30.4	0.53	0.83
Adjusted Final BW^8^, kg	581	578	4.4	0.47
*USDA Yield grade, %*				
YG 1, %	8.7	9.2	-	0.88
YG 2, %	27.8	28.7		
YG 3, %	54.4	53.5		
YG 4, %	7.5	7.2		
YG 5, %	1.6	1.5		
*USDA Quality grade, %*				
Standard, %	0.4	0.6	-	0.20
Select, %	19.7	29.7		
Low Choice, %	60.4	57.3		
Average Choice, %	15.0	9.6		
High Choice, %	3.2	1.9		
Prime, %	1.4	0.8		
*Liver score* ^*9*^				
Normal, %	60.4	66.2	-	0.55
A-, %	10.8	9.9		
A, %	6.7	5.9		
A + or greater, %	22.1	18.1		

^1^Treatments were applied from d 1 to d 46. DRC/DRC: received the DRC diet during the entire study; DRRG/DRC: received the DRRG diet during the initial 46 d, then fed to the DRC diet.

^2^Standard error of the mean (n = 7 pens/treatment).

^3^Observed significance for the effect of treatment.

^4^Calculated as: (HCW /Final BW × 0.96) × 100.

^5^Small00 = 400 (minimum for USDA Choice), Modest00 = 500 (minimum for USDA Average Choice).

^6^Empty body fat (Guiroy et al., 2002).

^7^Final BW, adjusted at 28% EBF (Guiroy et al., 2002).

^8^Determined based on the Elanco Liver Scoring System.

### Experiment 2

Growth performance data for Exp. 2 are presented in Table 6. Replacing 31.8% of the corn grain with hybrid RG during the entire finishing period (119 d) tended to reduce DMI expressed as % of BW (*P* = 0.09; 2.35 vs. 2.25 % of BW, for DRC vs. MIX, respectively), but did not affect DMI when expressed in absolute terms (*P* = 0.22). No other differences between treatment groups were observed for FBW, AFBW, ADG, G:F, paNEm, paNEg, and the ratios of O/E NEm and NEg (*P* ≥ 0.17).

**Table 6.** Growth performance of finishing steers fed either a corn-based diet or a mix of corn and rye-based diet for 119 d (Exp. 2)

	Treatment^1^			
	DRC	MIX	SEM^2^	*P*-value^3^
Steers, n	21	21	--	--
Pens, n	4	4	--	--
Initial BW (d 0), kg	433	440	2.2	--
Final BW (d 119), kg	659	658	11.3	0.97
Carcass adj. FBW^4^, kg	660	661	9.9	0.92
ADG, kg	1.90	1.83	0.090	0.66
DMI, kg	12.8	12.3	0.25	0.22
DMI, % of BW	2.35	2.25	5.87	0.09
G:F^5^, kg/kg	0.148	0.150	0.0048	0.83
F:G^6^, kg/kg	6.76	6.67	--	--
paNEm^7^, Mcal/kg	1.95	1.99	0.038	0.46
paNEg^7^, Mcal/kg	1.30	1.34	0.033	0.46
O/E NEm^8^	0.94	1.00	0.019	0.17
O/E NEg^8^	0.94	1.01	0.025	0.18

^1^For 119 d, steers received either a dry-rolled corn grain-based diet (DRC) or a mix of dry-rolled rye and corn-based diet (MIX).

^2^Standard Error of the mean (n = 4 pens/treatment).

^3^Observed significance for the effect of treatment.

^4^Calculated as: (HCW/Final BW × 0.96) × 100.

^5^Gain to feed, ADG/DMI.

^6^Feed to gain, reciprocal of G:F.

^7^Performance adjusted Net Energy for maintenance (paNEm) and gain (paNEg; Zinn et al., 2008).

^87^Observed to expected ratio for NEm and NEg; ratio of paNE values to tabular NE estimates from Preston (2017).

Carcass trait data for Exp. 2 are presented in Table 7. No differences were observed between treatments for any of the carcass attributes evaluated: HCW, DP, REA, RF, marbling score, yield score, EBF, and AFBW (*P* ≥ 0.24). Furthermore, no differences were observed between groups for the distributions of USDA Yield or Quality Grades, or percentage of normal livers (*P* ≥ 0.25).

**Table 7.** Carcass traits of beef steers fed either a dry-rolled corn-based or a mix of corn and rye-based diet for 119 d (Exp. 2)

	Treatment^1^			
	DRC	MIX	SEM^2^	*P*-value^3^
Hot carcass weight, kg	412	413	6.2	0.92
Dressing percentage^4^, %	62.8	62.9	0.33	0.92
Ribeye area, cm^2^	87.5	86.3	1.61	0.63
Rib fat, cm	1.39	1.47	0.037	0.24
Marbling score^5^	529	518	8.9	0.46
Yield Grade	3.48	3.63	0.083	0.31
EBF^6^, %	31.5	31.9	0.27	0.39
AFBW^7^, kg	589	584	6.2	0.64
* USDA Yield Grade, %*				
2	12.6	4.8		0.25
3	77.5	71.4		
4	8.9	21.1		
5	1.0	2.7		
* USDA Quality Grade, %*				
Select	5.49	8.2		0.54
Low Choice	30.27	37.9		
Upper 2/3rds Choice	58.87	50.4		
Prime	5.37	3.6		
* Liver abscess, %*				
No	98.9	99.2		0.85
Yes	1.1	0.8		

^1^For 119 d, steers received either a dry-rolled corn grain-based diet (DRC) or a mix of dry-rolled rye and corn-based diet (MIX).

^2^Standard Error of the mean (n = 4 pens/treatment).

^3^Observed significance for the effect of treatment.

^4^Calculated as: (HCW / FBW × 0.96) × 100.

^5^Small^00^ = 400 (minimum for USDA choice), Modest^00^ = 500 (minimum for USDA premium choice).

^6^EBF, empty body fat calculated (Guiroy et al., 2002).

^7^AFBW, final BW, adjusted to 28% EBF (Guiroy et al., 2002).

## DISCUSSION

A possible explanation for the reduced DMI observed in Exp. 1 during the initial days when DRRG was fed as the sole grain source was an increased risk for ruminal acidosis. Ruminal degradation of starch in RG is among the fastest among cereal grains. Krieg et al. (2017) observed in situ starch degradation rates of 116 vs. 85 and 36 %/h, and dry matter degradation rates of 82, 56, and 31%/h for rye, triticale, and barley, respectively. Similarly, in an in vitro experiment RG had greater dry matter digestibility and reduced pH compared to either dry-rolled or high-moisture corn (Rusche and Smith, 2024). Researchers at the University of Saskatchewan conducted a series of studies replacing barley grain with hybrid RG in beef finishing diets (Zhang et al., 2024a, 2024b). In their studies, increasing hybrid RG inclusions resulted in increased minutes per day with ruminal pH below 5.5, reduced DMI and ADG, reduced HCW, and increased liver abscess prevalence and severity (Zhang et al., 2024a, 2024b). Previous research demonstrated that feeding whole unprocessed hybrid RG as the sole source of cereal grain in finishing beef diets did not reduce DMI (Buckhaus et al., 2021). These observations support the position that the cause for reduced DMI in Exp. 1 while RG was the sole cereal grain source has more to do with starch degradation or sub-acute acidosis than RG inclusion per se.

Exposure to dietary EA has been shown to reduce DMI (Klotz, 2015; Coufal-Majewski et al., 2016; Sarich et al., 2023). Sarich et al. (2023) found that feeding diets with an EA concentration of 1.5 mg/kg reduced DMI in a finishing diet. However, feeding the same EA concentration in backgrounding diets did not affect DMI but reduced G:F. The mechanism by which EA can reduce DMI seems related to a reduction in gut motility. Ergot alkaloids can bind to the serotonergic receptors in smooth muscle tissue (Klotz, 2015). Consequently, reticulum contractions can be reduced (Poole et al., 2009), and ruminal arteries and veins can be contracted with the chance of a restriction in blood flow through the rumen wall (Foote et al., 2011). Sarich et al. (2023) suggested that the vascular effects of EA could reduce digestion, increase rumen fill, promote satiety, and reduce nutrient absorption across the rumen wall. However, in both our studies, the maximum dietary concentrations of EA in our diets (0.35 and 0.11 mg/kg, for Exp. 1 and 2, respectively) were less than the recommended upper limit for cattle diets of 0.5 mg/kg (Coufal-Majewski et al., 2016).

The disparity in observed DMI between the current experiment and previous work from our laboratory (Rusche et al., 2020; Buckhaus et al., 2021) when DRC was completely replaced by hybrid RG may be explained by differences in cattle management prior to initiation of the respective experiments. In our prior research evaluating complete replacement of DRC with hybrid RG, cattle used in those experiments had been consuming grazed forage prior to being shipped to feedlot facilities (Rusche et al., 2020; Buckhaus et al., 2021). The steers fed in Exp. 1 had been in a drylot facility consuming harvested feeds prior to being shipped to SERF and likely experienced a greater caloric intake compared to cattle grazing pasture in late summer. Nutritional status can influence food preference and avoidance (Forbes and Kyriazakis, 1995; Provenza, 1995) and thus it is possible that the cattle coming from a lower plane of nutrition were more willing to consume a novel feedstuff compared to steers from Exp. 1 that had been fed a higher quality diet.

The reduction in DMI during the replacement phase of Exp. 1 for the DRRG-fed steers led to less ADG and apparent net energy of the diet during this period, resulting in differing ratios between observed and expected NE values (Zinn et al., 2008; Owens and Hicks, 2019). After the steers were switched to the DRC-based diet they exhibited compensatory gain as there were no differences for final body weight at the end of the study. Nonetheless, this initial period of reduced gain for steers on Exp.1 may be the reason for the observed tendency to reduce marbling score and yield grade for the steers initially fed the DRRG-based diet. Similarly, in the study by Zhang et al. (2024a), dietary inclusions of 29% RG resulted in similar DMI and overall carcass characteristics as the barley grain control-fed animals, while greater inclusions of RG (59 and 88%) reduced DMI, ADG, final BW, Marbling percentage and yield score. Correspondingly, in our previous study, a 66 or 100% replacement of DRC with DRRG led to reduced DMI, ADG, GF, apparent NEg, and HCW (Rusche et al., 2020). In the same study, a 33% replacement of DRC with DRRG did not affect any of the mentioned parameters (Rusche et al., 2020). The rate of gain at an early stage of the feeding period has been shown to impact the carcass characteristics of beef steers. Steers that were limit-fed during the initial 56 or 86 d on feed had reduced fat thickness and marbling scores compared to steers fed ad libitum for the entire finishing period (Delver et al., 2024). Similarly, Blom et al. (2022) found that the rate of gain during the backgrounding period can have carryover effects on carcass characteristics of beef cattle finished with the same diet.

The lack of differences in growth performance or carcass characteristics when DRRG replaced 31% of DRC in the diet (Exp. 2) is aligned with the lack of differences in DMI. Previous work where DRRG replaced 33% of either DRC (Rusche et al., 2020) or barley (Zhang et al., 2024a) resulted in minimal impacts on performance and carcass characteristics. The results of the two experiments described here, as well as that previously reported (Rusche et al., 2020; Zhang et al., 2024a) support the conclusion that offering hybrid RG at greater than 20% of diet DM depresses feed intake. Further research should be conducted to determine the mechanisms for this intake reduction and whether there are mitigation strategies that could be implemented to overcome these limitations. Collectively, these data support limiting hybrid RG inclusion in finishing beef diets to optimize cattle performance and feed efficiency.

## CONCLUSION

In conclusion, a partial replacement of DRC with DRRG during the 119 d of the finishing period did not affect growth performance or carcass characteristics. By contrast, a total replacement during the initial 46 d negatively impacted intake and growth performance, with a recovery from the animals when they switched back to DRC. However, the initial total replacement had detrimental effects on marbling and yield grade. Partial replacement of corn with hybrid rye during the entire feeding optimized use of this feedstuff without sacrificing cattle performance.

## References

[CIT0001] AOAC. 2012. Official methods of analysis. 19th ed. Arlington (VA): Association of the Official Analytical Chemist.

[CIT0002] AOAC. 2016. Official methods of analysis of AOAC International. 20th ed. Arlington (VA): Association of the Official Analytical Chemist.

[CIT0003] Blom, E. J., W. W. Gentry, R. H. Pritchard, and K. E. Hales. 2022. Effects of backgrounding-phase rate of gain on performance and carcass characteristics of feedlot steers. Applied Animal Science 38:279–284. doi: https://doi.org/10.15232/aas.2022-02274

[CIT0004] Bowles, T. M., M. Mooshammer, Y. Socolar, F. Calderon, M. A. Cavigelli, S. W. Culman, W. Deen, C. F. Drury, A. Garcia y Garcia, and A. C. M. Gaudin. 2020. Long-term evidence shows that crop-rotation diversification increases agricultural resilience to adverse growing conditions in North America. One Earth 2:284–293. doi: https://doi.org/10.1016/j.oneear.2020.02.007

[CIT0005] Bowley, S. R. 2015. A Hitchhiker’s Guide to Statistics in Biology – Generalized Linear Mixed Model Edition. Guelph (ON): Any Old Subject Books.

[CIT0006] Brown, T. R., and T. E. Lawrence. 2010. Association of liver abnormalities with carcass grading performance and value. J. Anim. Sci. 88:4037–4043. doi: https://doi.org/10.2527/jas.2010-3219 20817855

[CIT0007] Buckhaus, E. M., W. C. Rusche, and Z. K. Smith. 2021. Effect of complete replacement of dry-rolled corn with unprocessed rye on growth performance, efficiency of dietary net energy use, and carcass traits of finishing heifers. Animals 11:99. doi: https://doi.org/10.3390/ani11010099 33419121 PMC7825491

[CIT0008] Coufal-Majewski, S., K. Stanford, T. McAllister, B. Blakley, J. McKinnon, A. V. Chaves, and Y. Wang. 2016. Impacts of cereal ergot in food animal production. Front. Vet. Sci 3:15. doi: https://doi.org/10.3389/fvets.2016.00015 26942186 PMC4766294

[CIT0009] Dahlke, G., B. Doran, S. Hoyer, and L. Schulz. 2021. Benchmarking the performance of Iowa feedlotcattle. Iowa St. Univ. Ames, IA (USA). IBC 0144. [Accessed April 3, 2023]. https://store.extension.iastate.edu/Product/16292

[CIT0010] Delver, J. J., W. C. Rusche, L. R. Thompson, F. L. Francis, B. B. Grimes Francis, R. J. Leeson, T. L. Maia Ribeiro, G. H. Olinger, C. R. Ross, and Z. K. Smith. 2024. Evaluating the effect limit feeding yearling beef steers for 56 or 86 d of a 140-d finishing period on growth performance and carcass trait responses. J. of Anim. Sci. 102(Suppl. 3):361–362. doi: https://doi.org/10.1093/jas/skae234.412

[CIT0011] Foote, A. P., D. L. Harmon, J. R. Strickland, L. P. Bush, and J. L. Klotz. 2011. Effect of ergot alkaloids on contractility of bovine right ruminal artery and vein. J. Anim. Sci. 89:2944–2949. doi: https://doi.org/10.2527/jas.2010-3626 21512122

[CIT0012] Forbes, J. M., and I. Kyriazakis. 1995. Food preferences in farm animals: why don’t they always choose wisely? Proc. Nutr. Soc. 54:429–440. doi: https://doi.org/10.1079/pns19950012 8524890

[CIT0013] Gaudet D. , Menzies J., Burnett P. 2000. Smuts, Bunts and Ergot. Encyclopedia of Microbiology. 4. 2nd ed. San Diego, CA (USA): Academic Press. p. 297–315.

[CIT0014] Goering, H. K., and P. J. VanSoest. 1970. Forage fiber analyses (apparatus, reagents, procedures, and some applications), Agric. Handbook No. 379. Washington (DC): ARS, USDA.

[CIT0015] Guiroy, P. J., L. O. Tedeschi, D. G. Fox, and J. P. Hutcheson. 2002. The effects of implant strategy on finished body weight of beef cattle. J. Anim. Sci. 80:1791–1800. doi: https://doi.org/10.2527/2002.8071791x 12162646

[CIT0016] Hansen, H. B., B. Møller, S. B. Andersen, J. R. Jørgensen, and A. Hansen. 2004. Grain characteristics, chemical composition, and functional properties of rye (Secale cereale L.) as influenced by genotype and harvest year. J. Agric. Food Chem. 52:2282–2291. doi: https://doi.org/10.1021/jf0307191 15080634

[CIT0017] Klotz, J. L. 2015. Activities and effects of ergot alkaloids on livestock physiology and production. Toxins 7:2801–2821. doi: https://doi.org/10.3390/toxins7082801 26226000 PMC4549725

[CIT0018] Krieg, J., N. Seifried, H. Steingass, and M. Rodehutscord. 2017. In situ and in vitro ruminal starch degradation of grains from different rye, triticale and barley genotypes. Animal 11:1745–1753. doi: https://doi.org/10.1017/S1751731117000337 28219468

[CIT0019] Lofgreen, G. P., and W. N. Garrett. 1968. A system for expressing net energy requirements and feed values for growing and finishing beef cattle. J. Anim. Sci. 27:793. doi: https://doi.org/10.2527/jas1968.273793x

[CIT0020] Menzies, J. G., and T. K. Turkington. 2015. An overview of the ergot (Claviceps purpurea) issue in western Canada: Challenges and solutions. Can. J. Plant. Path. 37:40–51. doi: https://doi.org/10.1080/07060661.2014.986527

[CIT0021] NASEM. 1996. Nutrient requirements of beef cattle. 7th ed. Washington (DC): The National Academies Press.

[CIT0022] NASEM. 2016. Nutrient requirements of beef cattle. 8th rev. ed. Washington, DC: Natl. Acad. Press,. doi: https://doi.org/10.17226/19014

[CIT0023] Owens, F. N., and R. B. Hicks. 2019. Can net energy values be determined from animal performance measurements? A review of factors affecting application of the California Net Energy System. Transl Anim Sci 3:929–944. doi: https://doi.org/10.1093/tas/txy130 32704857 PMC7252571

[CIT0024] Poffenbarger, H., G. Artz, G. Dahlke, W. Edwards, M. Hanna, J. Russell, H. Sellers, and M. Liebman. 2017. An economic analysis of integrated crop-livestock systems in Iowa, U.S.A. Agric. Sys. 157:51–69. doi: https://doi.org/10.1016/j.agsy.2017.07.001

[CIT0025] Poole, D. P., R. A. Littler, B. L. Smith, and L. M. McLeay. 2009. Effects and mechanisms of action of the ergopeptides ergotamine and ergovaline and the effects of peramine on reticulum motility of sheep. Am. J. Vet. Res. 70:270–276. doi: https://doi.org/10.2460/ajvr.70.2.270 19231961

[CIT0026] Preston, R. L. 2017. Feed composition table. https://www.beefmagazine.com/cattle-nutrition/feed-composition-tables-how-to-use-the-2017-data-to-mix-the-best-feed-for-your-cattle

[CIT0027] Provenza, F. D. 1995. Postingestive feedback as an elementary determinant of food preference and intake in ruminants. J. Range Manag. 48:2–17. doi: https://doi.org/10.2307/4002498

[CIT0028] Rusche, W. C., and Z. K. F. Smith. 2024. Evaluation of ruminal dry matter disappearance and pH of dry corn, high-moisture corn, and rye under *in vitro* conditions. Agric. Sci. 15:327–332. doi: https://doi.org/10.4236/as.2024.153019

[CIT0029] Rusche, W. C., J. A. Walker, P. Sexton, R. S. Brattain, and Z. K. Smith. 2020. Evaluation of hybrid rye on growth performance, carcass traits, and efficiency of net energy utilization in finishing steers. Trans. Anim. Sci 4:1–10. doi: https://doi.org/10.1093/tas/txaa173 PMC7592850 33134876

[CIT0030] Sarich, J. M., K. Stanford, K. S. Schwartzkopf-Genswein, T. A. McAllister, B. R. Blakley, G. B. Penner, and G. O. Ribeiro. 2023. Effect of increasing concentration of ergot alkaloids in the diet of feedlot cattle: performance, welfare, and health parameters. J. Anim. Sci. 101:skad287. doi: https://doi.org/10.1093/jas/skad287 37638650 PMC10506379

[CIT0031] USDA. 2017. United States Standards for Grades of Carcass Beef. Washington, DC: Agricultural Marketing Service.

[CIT0032] WHO. 2006. Safety evaluation of certain contaminants in food. Prepared by the Sixty-fourth meeting of the Joint FAO/WHO Expert Committee on Food Additives (JECFA), February 8-17, 2005. FAO food and nutrition paper. Geneva, Switzerland. 82:1–778. 17340910

[CIT0033] Zhang, F., R. E. Carey, R. S. Brattain, H. Wehrle, and G. B. Penner. 2024a. Production and use of dry-rolled hybrid rye grain as a replacement for barley grain on growth performance and carcass quality of feedlot steers. Transl Anim Sci 8:txae059. doi: https://doi.org/10.1093/tas/txae059 38707258 PMC11067788

[CIT0034] Zhang, F., R. E. Carey, R. S. Brattain, H. Wehrle, and G. B. Penner. 2024b. Effects of feeding hybrid rye grain as a replacement for barley grain on dry matter intake, ruminal fermentation, and the site and extent of nutrient digestion in finishing beef heifers. J. Anim. Sci. 102:skae211. doi: https://doi.org/10.1093/jas/skae211 39051129 PMC11329801

[CIT0035] Zinn, R. A., A. Barreras, F. N. Owens, and A. Plascencia. 2008. Performance by feedlot steers and heifers: Daily gain, mature body weight, dry matter intake, and dietary energetics. J. Anim. Sci. 86:2680–2689. doi: https://doi.org/10.2527/jas.2007-0561 18539825

[CIT0036] Zinn, R. A., and Y. Shen. 1998. An evaluation of ruminally degradable intake protein and metabolizable amino acid requirements of feedlot calves. J. Anim. Sci. 76:1280–1289. doi: https://doi.org/10.2527/1998.7651280x 9621934

